# An implementation of normal distribution based segmentation and entropy controlled features selection for skin lesion detection and classification

**DOI:** 10.1186/s12885-018-4465-8

**Published:** 2018-06-05

**Authors:** M. Attique Khan, Tallha Akram, Muhammad Sharif, Aamir Shahzad, Khursheed Aurangzeb, Musaed Alhussein, Syed Irtaza Haider, Abdualziz Altamrah

**Affiliations:** 1 Department of Computer Science, COMSATS Institute of Information Technology, Wah, Pakistan; 2 Department of Electrical Engineering, COMSATS Institute of Information Technology, Wah, Pakistan; 3 Department of Electrical Engineering, COMSATS Institute of Information Technology, Abbottabad, Pakistan; 4 College of Computer and Information Sciences, King Saud University, Riyadh, Saudi Arabia; 5 Department of Electrical Engineering, COMSATS Institute of Information Technology, Attock, Pakistan

**Keywords:** Image enhancement, Uniform distribution, Image fusion, Multi-level features extraction, Features fusion, Features selection

## Abstract

**Background:**

Melanoma is the deadliest type of skin cancer with highest mortality rate. However, the annihilation in its early stage implies a high survival rate therefore, it demands early diagnosis. The accustomed diagnosis methods are costly and cumbersome due to the involvement of experienced experts as well as the requirements for the highly equipped environment. The recent advancements in computerized solutions for this diagnosis are highly promising with improved accuracy and efficiency.

**Methods:**

In this article, a method for the identification and classification of the lesion based on probabilistic distribution and best features selection is proposed. The probabilistic distribution such as normal distribution and uniform distribution are implemented for segmentation of lesion in the dermoscopic images. Then multi-level features are extracted and parallel strategy is performed for fusion. A novel entropy-based method with the combination of Bhattacharyya distance and variance are calculated for the selection of best features. Only selected features are classified using multi-class support vector machine, which is selected as a base classifier.

**Results:**

The proposed method is validated on three publicly available datasets such as PH2, ISIC (i.e. ISIC MSK-2 and ISIC UDA), and Combined (ISBI 2016 and ISBI 2017), including multi-resolution RGB images and achieved accuracy of 97.5%, 97.75%, and 93.2%, respectively.

**Conclusion:**

The base classifier performs significantly better on proposed features fusion and selection method as compared to other methods in terms of sensitivity, specificity, and accuracy. Furthermore, the presented method achieved satisfactory segmentation results on selected datasets.

## Background

Skin cancer is reported to be one of the most rapidly spreading cancer amongst other types. It is broadly classified into two primary classes; Melanoma and Benign. The Melanoma is the deadliest type of cancer with highest mortality rate worldwide [1]. In the US alone, an astonishing mortality rate of 75% is reported due to melanoma compared to other types of skin cancers [2]. The occurrence of melanoma reported to be doubled (increases 2 to 3% per year) in the last two decades, faster than any other types of cancer. American Cancer Society (ACS) has estimated, 87,110 new cases of melanoma will be diagnosed and 9,730 people will die in the US only in 2017 [3]. Malignant melanoma can be cured if detected at its early stages, e.g., if diagnosed at stage I, the possible survival rate is 96%, compared to 5% at its stage IV [4, 5]. However, early detection is strenuous due to its high resemblance with benign cancer, even an expert dermatologist can diagnose it wrongly. A specialized technique of dermatoscopy is mostly followed by dermatologist to diagnose melanoma. In a clinical examination, most commonly adopted methods of visual features inspection are; Menzies method [6], ABCD rule [7], and 7-point checklist [8]. The most commonly used methods are the ABCD (atypical, border, color, diameter) rules and pattern analysis. It is reported that this traditional dermoscopy method can increase the detection rate 10 to 27% [9]. These methods distinctly increases the detection rate compared to conventional methods but still dependent on dermatologist’s skills and training [10]. To facilitate experts numerous computerized analysis systems have been proposed recently [11, 12] which are referred to as pattern analysis/ computerized dermoscopic analysis systems. These methods are non-invasive and image analysis based technique to diagnose the melanoma.

In the last decade, several non-invasive methods were introduced for the diagnosis of melanoma including optical imaging system (OIS) [13], optical coherence tomography (OCT) [14], light scattering (LS) [15], spectropolarimetric imaging system (SIM) [16, 17], fourier polarimetry (FP) [18], polarimetric imaging [19], reectance confocal microscopy (RCM) [20, 21], photo-acoustic microscopy [22], optical transfer diagnosis (OTD) [23], etc. All these above mentioned methods have enough potential to diagnose the skin lesions and also accurate enough to distinguish the melanoma and benign. The optical methods are mostly utilized during a clinal tests to evaluate the presurgical boundaries of the basal cell carcinoma. It can help in drawing boundaries around the region of interest (ROI) in the dermoscopic images. LS skin methods give the information about the micro-architecture, which is represented with small pieces of pigskin and mineral element and helps to determine the extent of various types of skin cancers. The SIM method correctly evaluates the polarimetric contrast of the region of interest or infectious region such as melanoma, compared to the background or healthy region. However, in FP method human skins is observed with laser scattering and difference is identified using optical method for the diagnostic test for differentiating melanoma and benign.

### Problem statement

It is proved that malignant melanoma is a lethal skin cancer that is extra dominant between the 15 and above aged people [24]. The recent research shows high rate of failure to detect and diagnose this type of cancer at the early stages [25]. Generally, it consists of four major steps: preprocessing, which consists of hair removal, contrast enhancement, segmentation, feature extraction, and finally classification. The most challenging task in dermoscopy is an accurate detection of lesion’s boundary because of different artifacts such as hairs, illumination effects, low lesion contrast, asymmetrical and irregular border, nicked edges, etc. Therefore, for an early detection of melanoma, shape analysis is more important. In features extraction step, several types of features are extracted such as shape, color, texture, local etc. But, we have no clear knowledge about salient features for classification.

### Contribution

In this article, we propose a new method of lesion detection and classification by implementing probabilistic distribution based segmentation method and conditional entropy controlled features selection. The proposed technique is an amalgamation of five major steps: a) contrast stretching; b) lesion extraction; c) multi-level features extraction; d) features selection and e) classification of malignant and benign. The results are tested on three publicly available datasets which are PH2, ISIC (i.e. ISIC MSK-2 and ISIC UDA), and Combined (ISBI 2016 and ISBI 2017), containing RGB images of different resolutions, which are later normalized in our proposed technique. Our main contributions are enumerated below:

1. Enhanced the contrast of a lesion area by implementing a novel contrast stretching technique, in which we first calculated the global minima and maxima from the input image and then utilized low and high threshold values to enhance the lesion.
2. Implemented a novel segmentation method based on normal and uniform distribution. Mean of the uniform distribution is calculated from the enhanced image and the value is added in an activation function, which is introduced for segmentation. Similarly, mean deviation of the normal distribution is calculated using enhanced image and also inserted their values in an activation function for segmentation.
3. A fusion of segmented images is implemented by utilizing additive law of probability.
4. Implemented a novel feature selection method, which initially calculate the Euclidean distance between fused feature vector by implementing an Entropy-variance method. Only most discriminant features are later utilized by multi-class support vector machine for classification.

### Paper organization

The chronological order of this article is as follows: The related work of skin cancer detection and classification is described in “Related work” section. “Methods” section explains the proposed method, which consists of several sub steps including contrast stretching, segmentation, features extraction, features fusion, classification etc. The experimental results and conclusion of this article are described in “Results” and “Discussion” sections.

## Related work

In the last few decades, advance techniques in different domains of medical image processing, machine learning, etc., have introduced tremendous improvements in computer aided diagnostic systems. Similarly, improvements in dermatological examination tools have led the revolutions in the prognostic and diagnostic practices. The computerized features extractions of cutaneous lesion images and features analysis by machine learning techniques have potential to enroute the conventional surgical excision diagnostic methods towards CAD systems.

In literature several methods are implemented for automated detection and classification of skin cancer from the dermoscopic images. Omer et al. [26] introduced an automated system for an early detection of skin lesion. They utilized color features prior to global thresholding for lesion’s segmentation. The enhanced image was later subjected to 2D Discrete Fourier Transform (DCT) and 2D Fast Fourier Transform (FFT) for features extraction prior to the classification step. The results were tested on a publicly available dataset PH2. Barata et al. [27] described the importance of color features for detection of skin lesion. The color sampling method is utilized with Harris detector and compared their performance with grayscale sampling. Also, compared the color-SIFT (scale invariant feature transform) and SIFT features and conclude that color-SIFT features performs good as compare to SIFT. Yanyang et al. [28] introduced an novel method for melanoma detection based on Mahalanobis distance learning and graph regularized non-negative matrix factorization. The introduced method treated as a supervised learning method and reduced the dimensionality of extracted set of features and improves the classification rate. The method is evaluated on PH2 dataset and achieved improved performance. Catarina et al. [29] described the strategy of combination of global and local features. The local features (BagOf Features) and global features (shape and geometric) are extracted from original image and fused these features based of early fusion and late fusion. The author claim the late fusion is never been utilized in this context and it gives better results as compared to early fusion.

Ebtihal et al. [30] introduced an hybrid method for lesion classification using color and texture features. Four moments such as mean standard deviation, degree of asymmetry and variance is calculated against each channel, which are treated as a features. The local binary pattern (LBP) and gray level co-occurrences matrices (GLCM) were extracted as a texture features. Finally, the combined features were classified using support vector machine (SVM). Agn et al. [31] introduced a saliency detection technique for accurate lesion detection. The introduced method resolve the problems when the lesion borders are vague and the contrast between the lesion and inundating skin is low. The saliency method is reproduced with the sparse representaion method. Further, a Bayesian network is introduced that better explains the shape and boundary of the lesion. Euijoon et al. [38] introduced a saliency based segmentation technique where the background of original image was detected by spatial layout which includes boundaries and color information. They implemented Bayesian framework to minimize the detection errors. Similarly, Lei et al. [32] introduced a new method of lesion detection and classification based on multi-scale lesion biased representation (MLR). This proposed method has the advantage of detecting the lesion using different rotations and scales, compared to conventional methods of single rotation.

From above recent studies, we noticed that the colour information and contrast stretching is an important factor for accurately detection of lesion from dermoscopic images. Since the contrast stretching methods improves the visual quality of lesion area and improves the segmentation accuracy. Additionally, for improved classification, several features are utilized in literature but according to best our knowledge, serial based features fusion is not yet utilized. However, in our case only salient features are utilized which are later subjected to fusion for improved classification.

## Methods

**Fig. 1:**
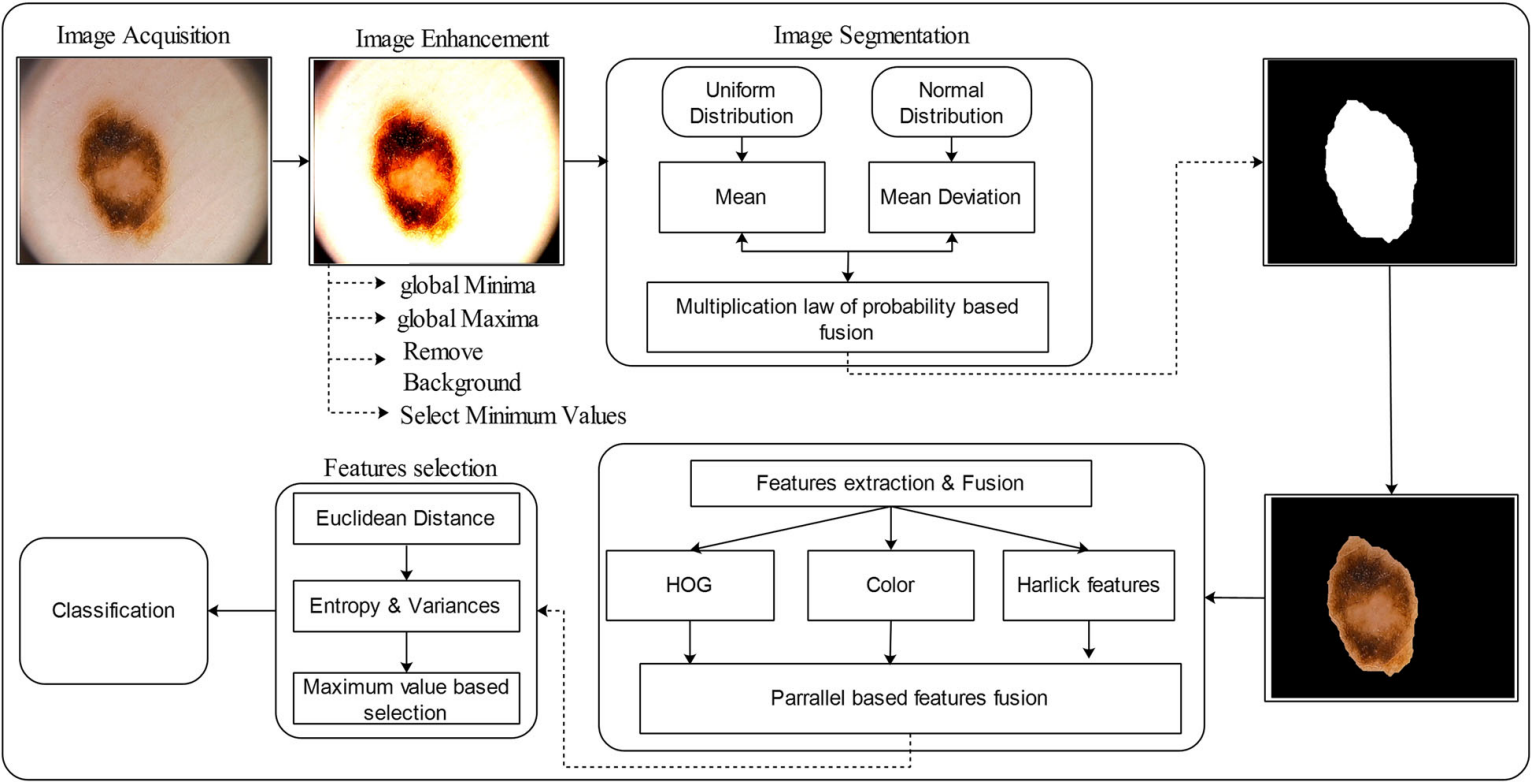
Proposed architecture of skin lesion detection and classification

A new method is proposed for lesion detection and classification using probabilistic distribution based segmentation method and conditional entropy controlled features selection. The proposed method is consists of two major steps: a) lesion identification; b) lesion classification. For lesion identification, we first enhance the contrast of input image and then segment the lesion by implementation of novel probabilistic distribution (uniform distribution, normal distribution). The lesion classification is done based of multiple features extraction and entropy controlled most prominent features selection. The detailed flow diagram of proposed method is shown in Fig. 1.

### Contrast stretching

There are numerous contrast stretching or normalization techniques [34], which attempt to improve the image contrast by stretching pixels’ specific intensity range to a different level. Most of the available options take gray image as an input and generate an improved output gray image. In our research work, the primary objective is to acquire a three channel *RGB* image having dimensions *m*×*n*×3. Although, the proposed technique can only work on a single channel of size *m*×*n*, therefore, in proposed algorithm we separately processed red, green and blue channel.

**Fig. 2:**
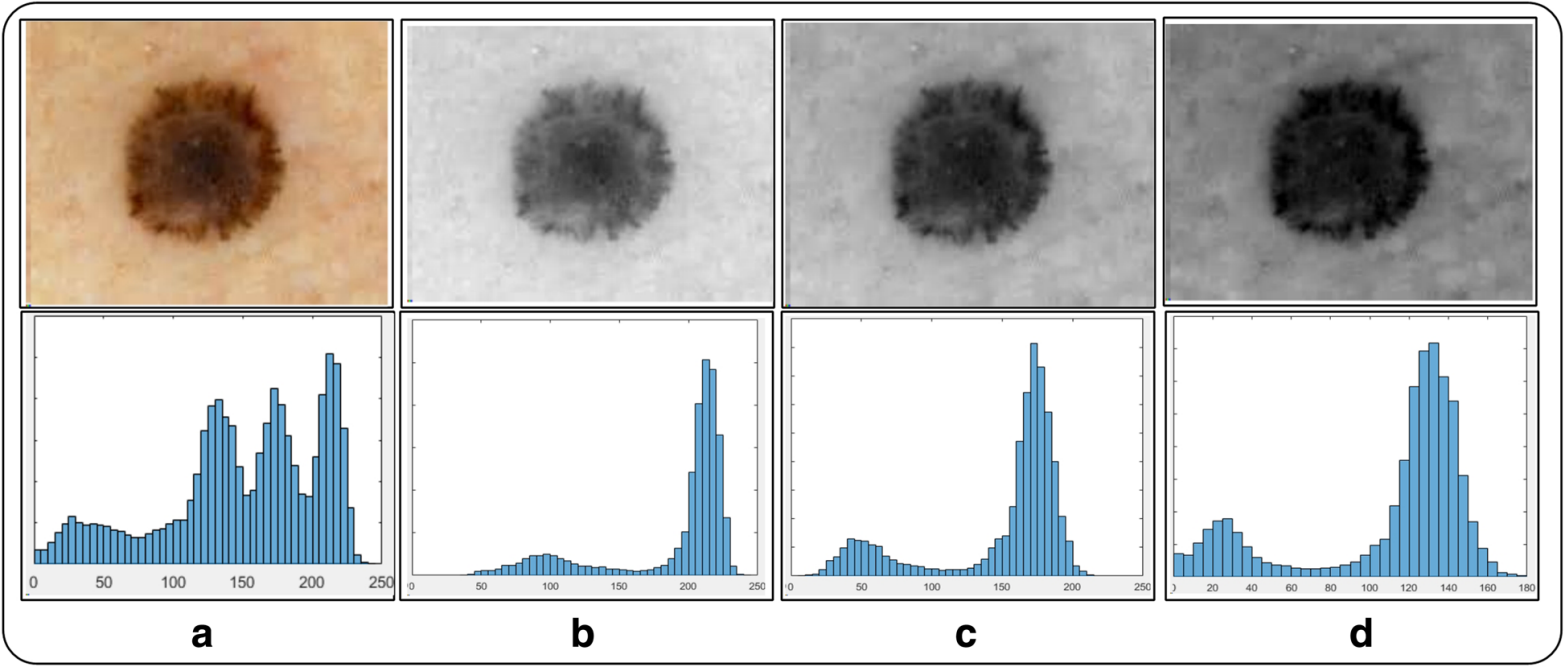
Information of original image and their respective channels: **a** original image; **b** red channel; **c** green channel; **d** blue channel

In *RGB* dermoscopic images, mostly the available contents are visually distinguishable into foreground which is infected region and the background. This distinctness is also evident in each and every gray channel, as shown in Fig. 2.

Considering the fact [35], details are always high with higher gradient regions which is foreground and details are low with the background due to low gradient values. We firstly divide the image into equal sized blocks and the compute weights for all regions and for each channel. For a single channel information, details are given below.

1. Gray channel is preprocessed using Sobel edge filter to compute gradients where kernel size is selected to be 3×3.
2. Gradient calculation for each equal sized block and rearranging in an ascending order. For each block the weights are assigned according to the gradient magnitude.
  1 $$ \Gamma\zeta(x,y) = \left\{\begin{array}{ll} \varsigma_{w}^{b1} & if \upsilon_{c}(x,y)\leq T_{1}; \\ \varsigma_{w}^{b2} & T_{1} < \upsilon_{c}(x,y)\leq T_{2}; \\ \varsigma_{w}^{b3} & T_{1} < \upsilon_{c}(x,y) \leq T_{3}; \\ \varsigma_{w}^{b4} & otherwise \\ \end{array}\right.  $$
  where $\varsigma _{w}^{bi}~(i\leq 4)$ are statistical weight coefficient and *T*_*i*_ is gradient intervals threshold.
3. Cumulative weighted gray value is calculated for each block using:
  2 $$ N_{g}(z)=\sum\limits_{i=1}^{4}\varsigma_{w}^{bi} n_{i}(z)  $$
  where *n*_*i*_(*z*) represents cumulative number of gray level pixels for each block *i*.
4. Concatenate red, green and blue channel to produce enhanced *RGB* image.

For each channel, three basic conditions are considered for optimized solution: I) extraction of regions with maximum information; II) selection of a block size; III) an improved weighting criteria. In most of the dermoscopic images, maximum informative regions are with in the range of 25−75*%*. Therefore, considering the minimum value of 25%, the number of blocks are selected to be 12 as an optimal number, with an aspect ratio of 8.3*%*. These blocks are later selected according to the criteria of maximal information retained (cumulative number of pixels for each block). Laplacian of Gaussian method (LOG) [36] is used with sigma value of two for edge detection. Weights are assigned according to the number of edge points, *E*_*pi*_ for each block:

3 $$ B_{wi}=\frac{E_{pi}}{E^{b}_{max}}  $$

where $E^{b}_{max}$ is the block with maximum edges. Finally, adjust the intensity levels of enhance image and perform log operation to improved lesion region as compare to original.

4 $$ \varphi(AI)=\zeta (B_{wi})  $$

5 $$ \varphi(t)=C \times log(\beta + \varphi(AI))  $$

**Fig. 3:**
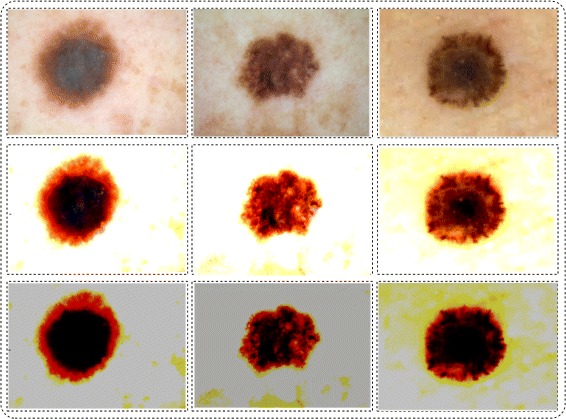
Proposed contrast stretching results

Where *β* is a constant value, (*β*≤10), which is selected to be 3 for producing most optimal results. *ζ* denotes the adjust intensity operation, *φ*(*AI*) is enhance image after *ζ* operation and *φ*(*t*) is final enhance image. The final contrast stretching results are shown in Fig. 3.

### Lesion segmentation

Segmentation of skin lesion is an important task in the analysis of skin lesions due to several problems such as color variation, presence of hairs, irregularity of lesion in the image and necked edges. Accurate segmentation provides important cues for accurate border detection. In this article, a novel method is implemented based of probabilistic distribution. The probabilistic distribution is consists of two major steps: a) uniform distribution based mean segmentation; b) normal distribution based segmentation.

#### Mean segmentation

The uniform distribution of mean segmentation is calculated from enhanced image *φ*(*t*) and then perform threshold function for lesion extraction. The detailed description of mean segmentation is defined below: Let *t* denotes the enhanced dermoscopic image and *f*(*t*) denotes the function of uniform distribution, which is determined as $f(t)=\frac {1}{y-x}$. Where *y* and *x* denotes the maximum and minimum pixels values of *φ*(*t*). Then the mean value is calculated as follows:

6 $$ \mu = \int_{x}^{y}t \ f(t)\ dt  $$

7 $$ \quad=\int_{x}^{y}t \ \frac{1}{y-x} \ dt  $$

8 $$ \quad=\frac{1}{y-x}\left [ \frac{t^{2}}{2} \right ]^{y}_{x}  $$

9 $$ \quad=\frac{1}{2(y-x)}\left [(y+x)(y-x) \right ]  $$

10 $$ \mu=\frac{1}{2}\left [(y+x) \right]  $$

**Fig. 4:**
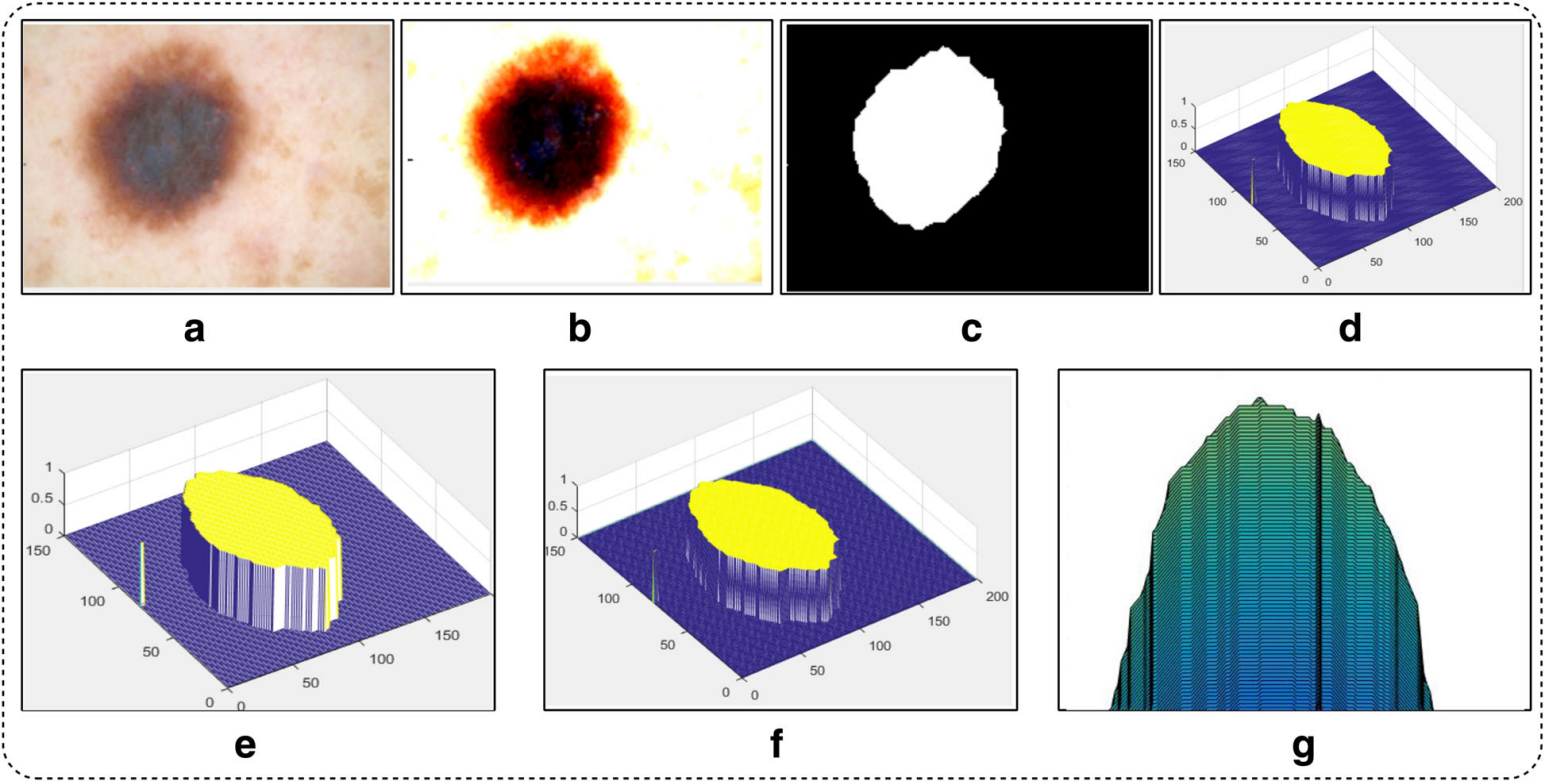
Proposed uniform distribution based mean segmentation results. **a** original image; **b** enhanced image; **c** proposed uniform based mean segmentation; **d** 2D contour image; **e** Contour plot; **f** 3D contour plot; **g** lesion area

Then perform an activation function, which is define as follows:

11 $$ A(\mu)=\frac{1}{\left (1+\left (\frac{\mu}{\varphi(t)} \right) \right)^{\alpha}}+\frac{1}{2\mu}+ C  $$

12 $$ F(\mu)=\left\{\begin{array}{ll} 1 & if\ A(\mu)\geq \delta_{thresh}\\ 0 & if\ A(\mu)<\delta_{thresh} \end{array}\right.  $$

where *δ*_*thresh*_ is Otus’s threshold, *α* is a scaling factor which controls the lesion area and its value is selected on the basis of simulations performed, *α*≤10, and finally got *α*=7 to be most optimal number. *C* is a constant value which is randomly initialized within the range of 0 to 1. The segmentation results are shown in Fig. 4.

#### Mean deviation based segmentation

The mean deviation (M.D) of normal distribution is is calculated from *φ*(*t*) having parameter *μ* and *σ*. The value of M.D is utilized by activation function for extraction of lesion from the dermoscopic images. Let *t* denotes the enhanced dermoscopic image and *f*(*t*) denotes the normalized function, which determined as $f(t)=\frac {1}{\sqrt {2\pi }\sigma }e^{-\frac {1}{2}(\frac {t-\mu }{\sigma })^{2}}$. Then initialize the M.D as:

13 $$ M.D=\int_{-\infty}^{+\infty}\left | t-\mu \right |f(t)  $$

14 $$ \qquad=\int_{-\infty}^{+\infty}\left | t-\mu \right | \frac{1}{\sqrt{2\pi}\sigma}e^{-\frac{1}{2}\left(\frac{t-\mu}{\sigma}\right)^{2}} dt  $$

Then put $g=\frac {t- \mu }{\sigma }$ in Eq. 14.

15 $$ M.D=\frac{1}{\sqrt{2\pi}\sigma}\int_{-\infty}^{+\infty}\left | \sigma g \right | e^{\frac{-g^{2}}{2}} dg  $$

16 $$ \qquad=\frac{\sigma}{\sqrt{2\pi}}\left [ \int_{0}^{\infty}g \ e^{\frac{-g^{2}}{2}} dg + \int_{0}^{\infty}g \ e^{\frac{-g^{2}}{2}} dg \right ]  $$

17 $$  M.D=\frac{2\sigma}{\sqrt{2\pi}} \int_{0}^{\infty}g \ e^{\frac{-g^{2}}{2}} dg  $$

Put $\frac {g^{2}}{2}=l$ in Eq. 17 and it becomes:

18 $$ M.D=\frac{2\sigma}{\sqrt{2\pi}} \int_{0}^{\infty}\sqrt{2l} \ e^{-l} \ \frac{dl}{\sqrt{2l}}  $$

19 $$ \qquad=\frac{2\sigma}{\sqrt{2\pi}} \int_{0}^{\infty} e^{-l} \ dl  $$

20 $$ \qquad=\sqrt{\frac{2}{\pi}}\sigma \left [ \frac{e^{-l}}{-1} \right ]^{\infty}_{0}  $$

21 $$ \qquad=-\sqrt{\frac{2}{\pi}}\sigma \left [ \frac{1}{e^{l}} \right ]^{\infty}_{0}  $$

22 $$ \qquad=-\sqrt{\frac{2}{\pi}}\sigma (-1)  $$

Hence

23 $$ M.D=0.7979 \sigma  $$

Then perform an activation function to utilize M.D as:

24 $$ AC(M.D)=\frac{1}{\left (1+\left (\frac{M.D}{\varphi(t)} \right) \right)^{\alpha}}+\frac{1}{2 \ M.D}+ C  $$

25 $$ F(M.D)=\left\{\begin{array}{ll} 1 & if\ AC(M.D)\geq \delta_{thresh}\\ 0 & if\ AC(M.D)< \delta_{thresh} \end{array}\right.  $$

**Fig. 5:**
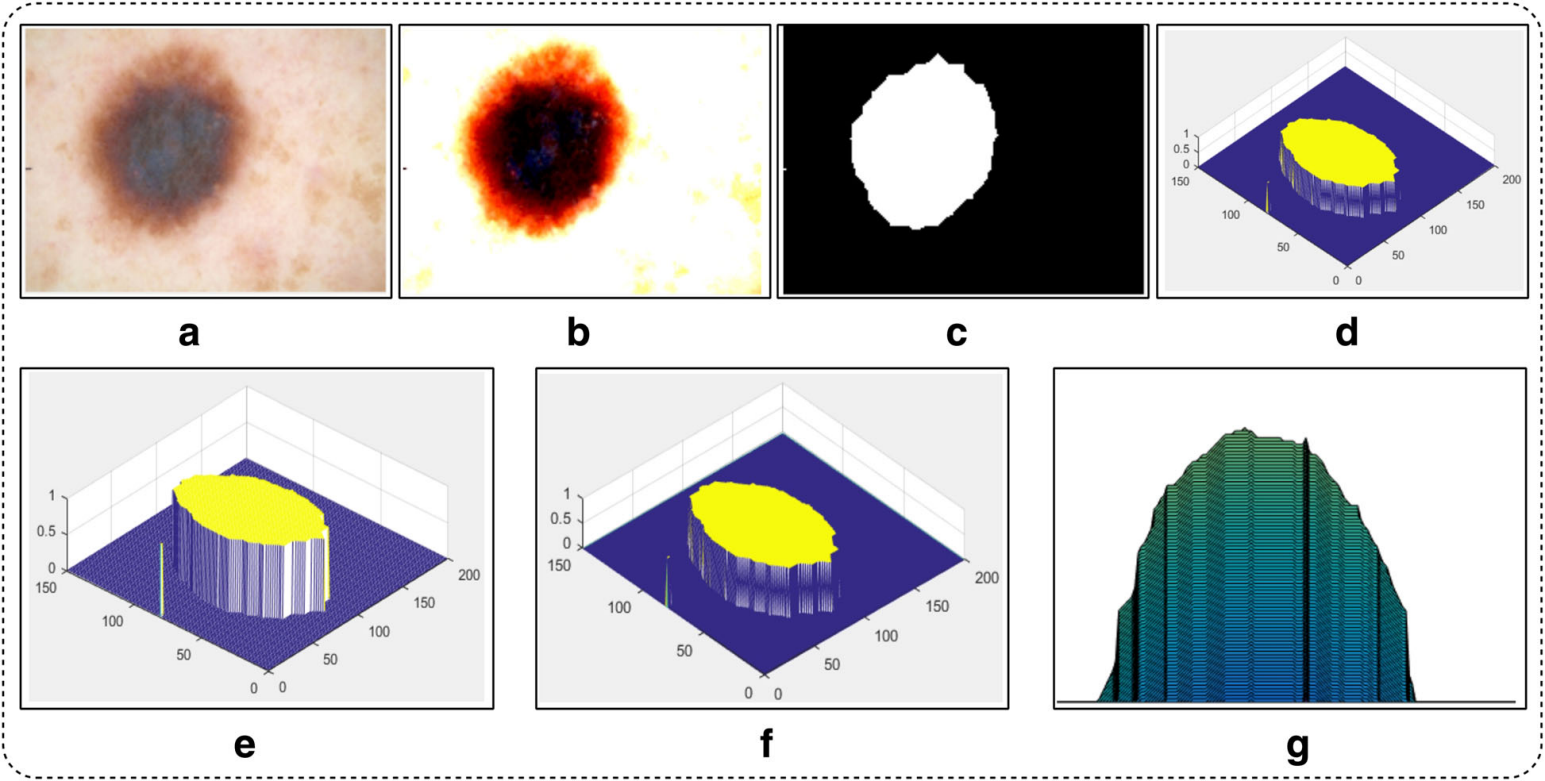
Proposed normal distribution based M.D segmentation results. **a** original image; **b** enhanced image; **c** proposed M.D based segmentation; **d** 2D contour image; **e** Contour plot; **f** 3D contour plot; **g** lesion area

The segmentation results of M.D is shown in Fig. 5.

#### Image fusion

The term image fusion mean to combine the information of two or more than two images in one resultant image, which contains better information as compare to any individual image or source. The image fusion reduces the redundancy between two or more images and increase the clinical applicability for diagnosis. In this work, we implemented a union based fusion of two segmented images into one image. The resultant image is more accurate and having much information as compare to individual. Suppose *N* denotes the sample space, which contains 200 dermoscopic images. Let *X*_1_∈*F*(*μ*) which is mean segmented image. Let *X*_2_∈*F*(*M*.*D*) which M.D based segmented image. Let *i* denotes the *X*_1_ pixels values and *j* denotes the *X*_2_ pixels values and *S* denotes the both *i* and *j* pixels which pixels values are 1. It mean all 1 value pixels fall in *S*. Then *X*_1_∪*X*_2_ written as:

26 $$ X_{1}\cup X_{2}=(X_{1} \cup X_{2})\cap \phi  $$

27 $$ P(X_{1}\cup X_{2})=P((X_{1} \cup X_{2}))\cap P(\phi)  $$

28 $$ =\left\{\begin{array}{lll} \xi((X_{1}, X_{2})==1) & if &(i,j) \in z_{1} \\ \xi((X_{1}, X_{2})==0) & if & (i,j) \in z_{2} \end{array}\right.  $$

Where *z*_1_ represented as ground truth Table 1.

**Table 1:** Ground truth table for *z*_1_

*X*_1_∈*i*	*X*_2_∈*j*	S
0	0	0
0	1	1
1	0	1
1	1	1

Hence

29 $$ \varrho (t)=\left\{\begin{array}{ll} 1 & if \ \ \sum\left[i, j\right]>1 \\ 0 & \ \ Otherwise \end{array}\right.  $$

30 $$ P(X_{1}\cup X_{2})=P(X_{1})+ P(X_{2})-P(\phi)  $$

**Fig. 6:**
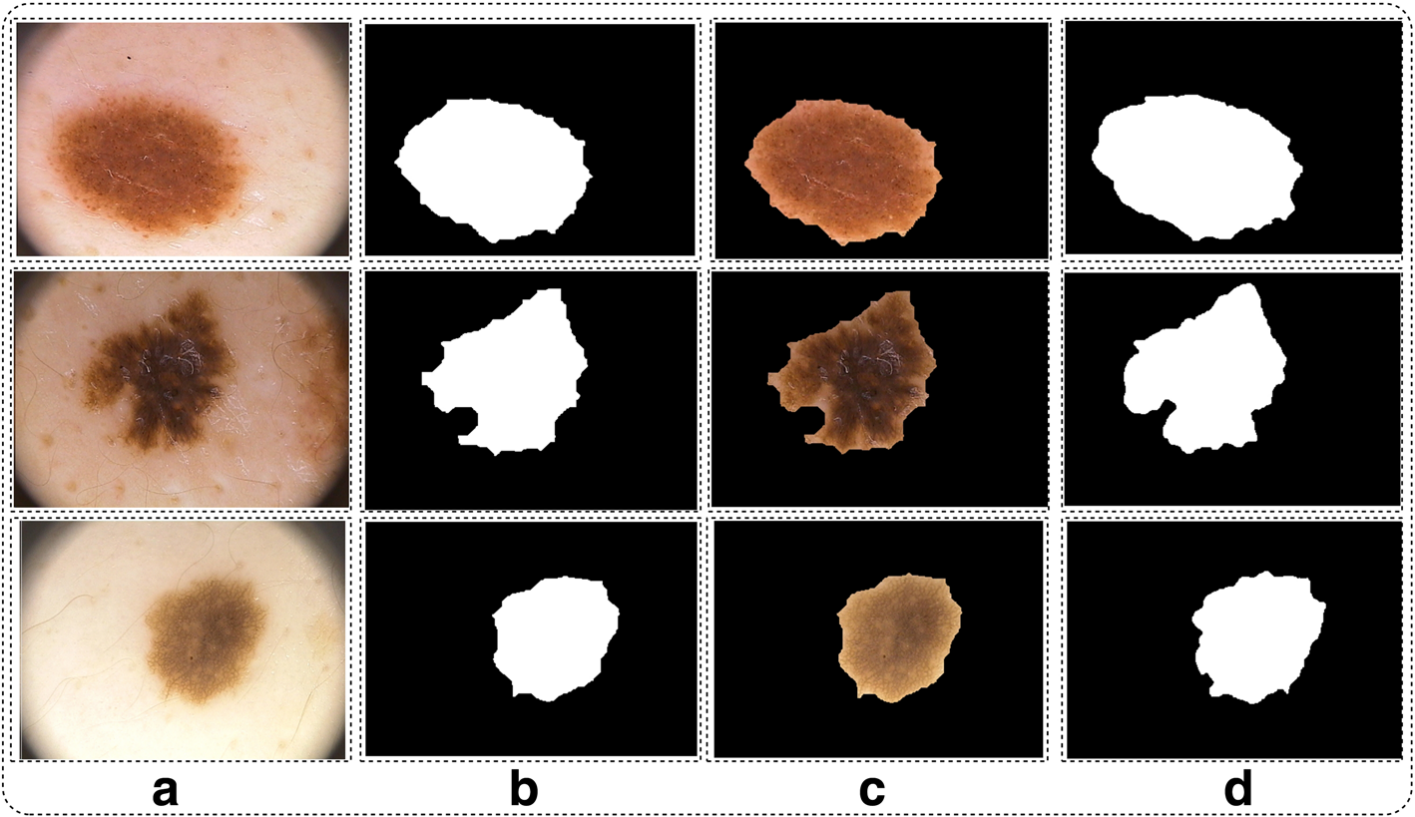
Proposed fusion results. **a** original image; **b** fused segmented image; **c** mapped on fused image; **d** ground truth image

Where *P*(*ϕ*) denotes the 0 values which presented as background and 1 denotes the lesion. The graphical results after fusion are shown in Fig. 6.

#### Analysis

**Fig. 7:**
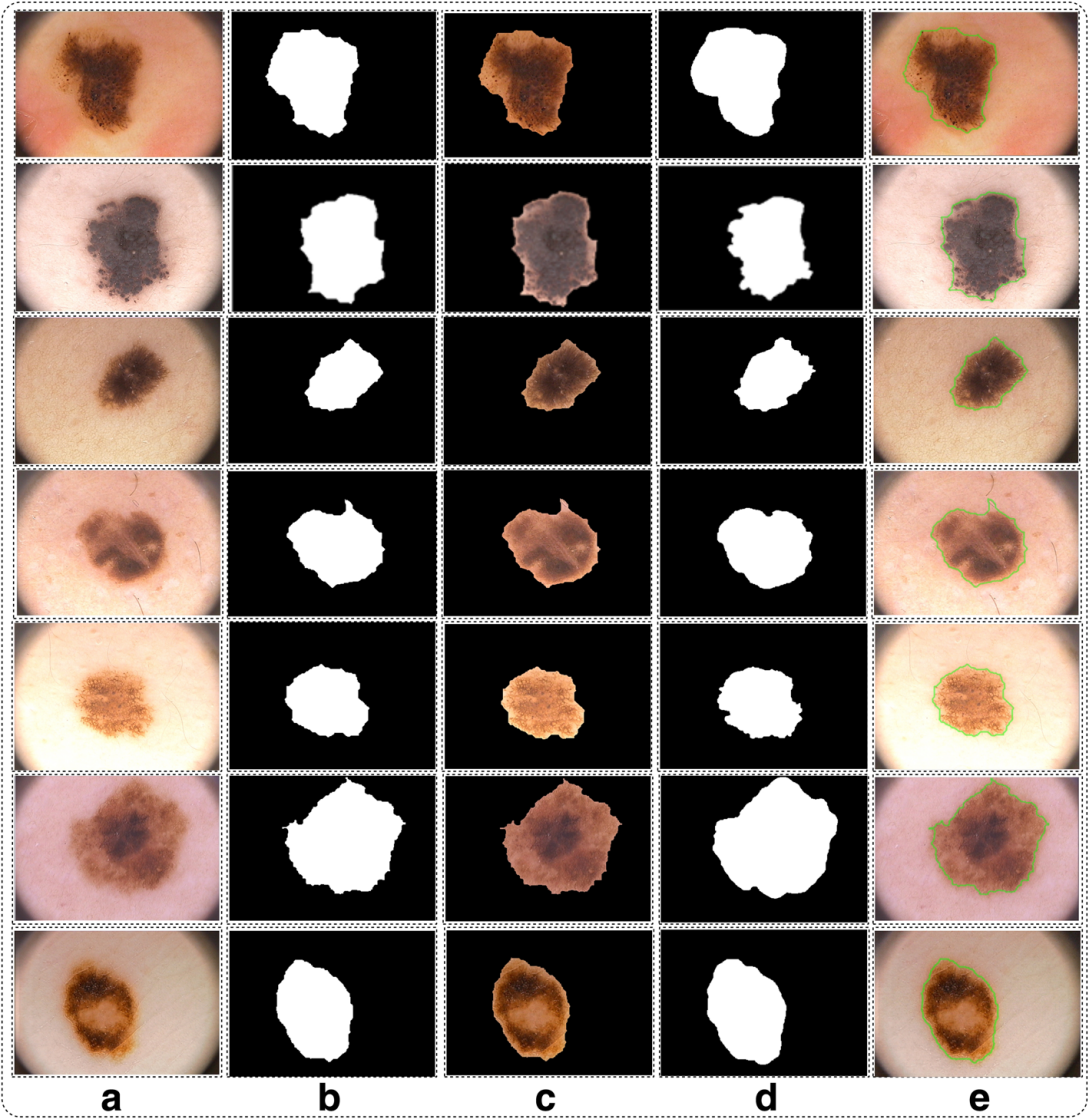
Proposed fusion results. **a** original image; **b** proposed segmented image; **c** mapped on proposed image; **d** ground truth image; **e** border on proposed segmented image

In this section, we analyze our segmentation results in terms of accuracy or similarity index as compared to given ground truth values. We select randomly images from PH2 dataset and shows their results in tabular and graphical. The proposed segmentation results are directly compare to ground truth images as shown in Fig. 7. The testing accuracy against each selected dermoscopic image are depicted in Table 2. From Table 2 the accuracy of each image is above 90% and the maximum similarity rate is 98.10. From our analysis, the proposed segmentation results perform well as compare to existing methods [31, 37–39] in terms of border detection rate.

**Table 2:** Lesion detection accuracy as compared to ground truth values

Image description	Similarity rate	Image description	Similarity rate
IMD038	95.69	IMD199	94.70
IMD020	92.52	IMD380	97.94
IMD039	91.35	IMD385	94.37
IMD144	88.33	IMD392	94.47
IMD203	86.44	IMD394	96.96
IMD379	88.41	IMD047	90.07
IMD429	94.87	IMD075	95.85
IMD211	92.81	IMD078	94.70
IMD285	95.59	IMD140	96.94
IMD022	96.02	IMD256	95.82
IMD025	96.35	IMD312	96.04
IMD042	91.26	IMD369	96.08
IMD173	96.04	IMD376	93.07
IMD182	97.97	IMD427	93.14
**IMD430**	**98.10**	IMD168	92.88

Data in bold are significant

### Image representation

In this three types of features are extracted for the representation of an input image. The basic purpose of feature extraction is to find out a combination of most efficient features for classification. The performance of dermoscopic images mostly depends on the quality and the consistency of the selected features. In this work, three types of features are extracted such as color, texture and HOG for classification of skin lesion.

#### HOG features

**Fig. 8:**
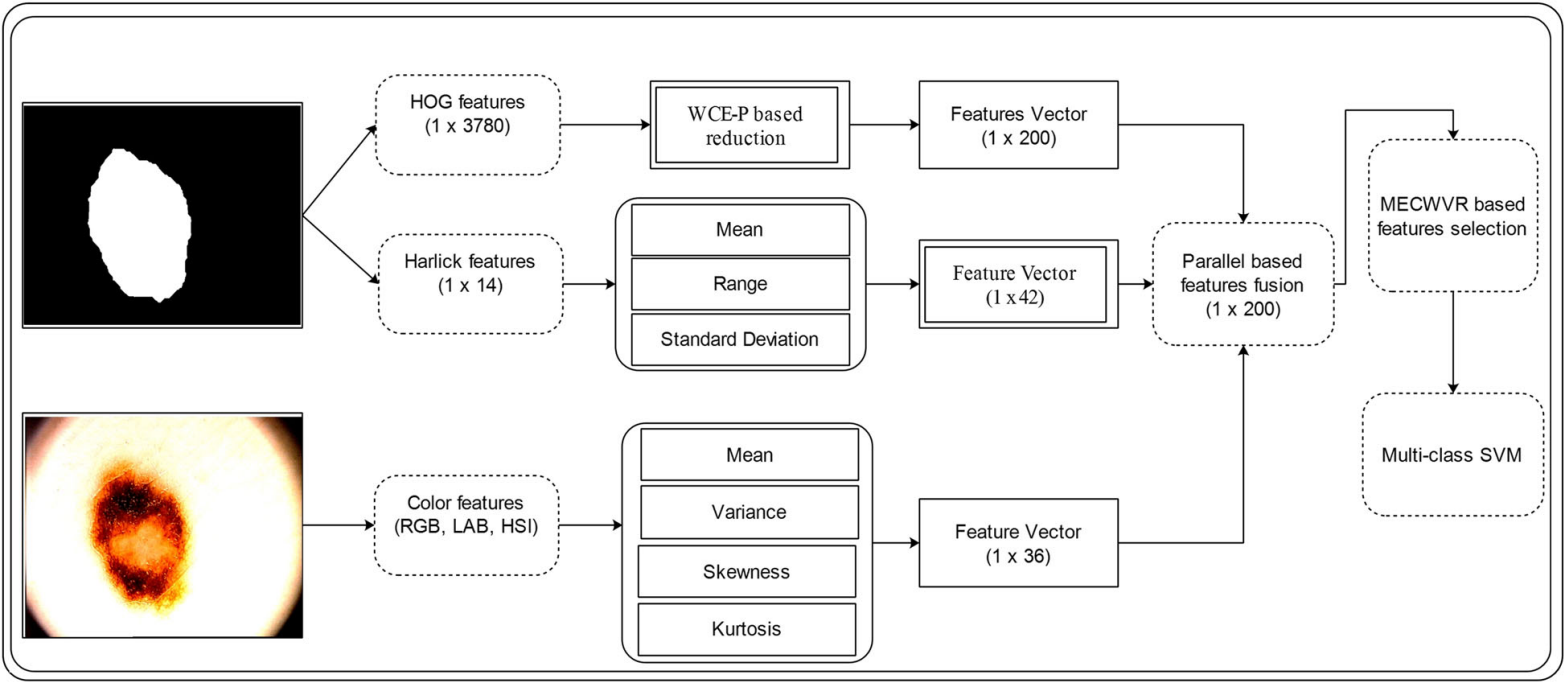
A system architecture of multiple features fusion and selection

The Histogram Oriented Gradients (HOG) features are originally introduced by Dalal [40] in 2005 for human detection. The HOG features are also called shape based features because they work on the shape of the object. In our case, the HOG features are extracted from segmented skin lesion and work efficiently because every segmented lesion have their own shape. As shown in Fig. 8, the HOG features are extracted from segmented lesion and obtain a feature vector of size 1×3780 because we have the size of segmented image is 96×128 and size of bins is 8×8. The size of extracted features are too high and they effect on the classification accuracy. For this reason, we implement a weighted conditional entropy with PCA (principle component analysis) on extracted feature vector. The PCA return the score against each feature and then weighted entropy is utilized to reduced the feature space and select the maximum 200 score features. The weighted conditional entropy is define as:

31 $$ E_{W}=\sum\limits_{i=1}^{K}\sum\limits_{j=1}^{K}W_{i,j}.\ P(i,j)log\frac{P(i)}{P(i,j)}  $$

Where *i*, *j* denotes the current and next feature respectively. *W*_*i*,*j*_ denotes the weights of selected features, which is selected between 0 and 1 (0≤*W*_*ij*_≤1) and $ P(i,j)=\frac {W_{ij}. \ n_{ij}}{\sum _{ij=1}^{K}W_{ij}. \ n_{ij}}$. Hence the new reduce vector size is 1×200.

#### Harlick features

Texture information of an input image is an important component, which is utilized to identify the region of interest such as a lesion. For texture information of lesion, we extract the harlick features [41]. The harlick features are extracted from the segmented image as shown in Fig. 8. There are total 14 texture features implemented (i.e. autocorrelation, contrast, cluster prominence, cluster shade, dissimilarity, energy, entropy, homogeneity 1, homogeneity 2, maximum probability, average, variances, inverse difference normalized and inverse difference moment normalized) and a feature vector of size 1×14 is created. After calculating the mean, range and variance of each feature, the final vector is calculated having size 1×42.

#### Color features

**Fig. 9:**
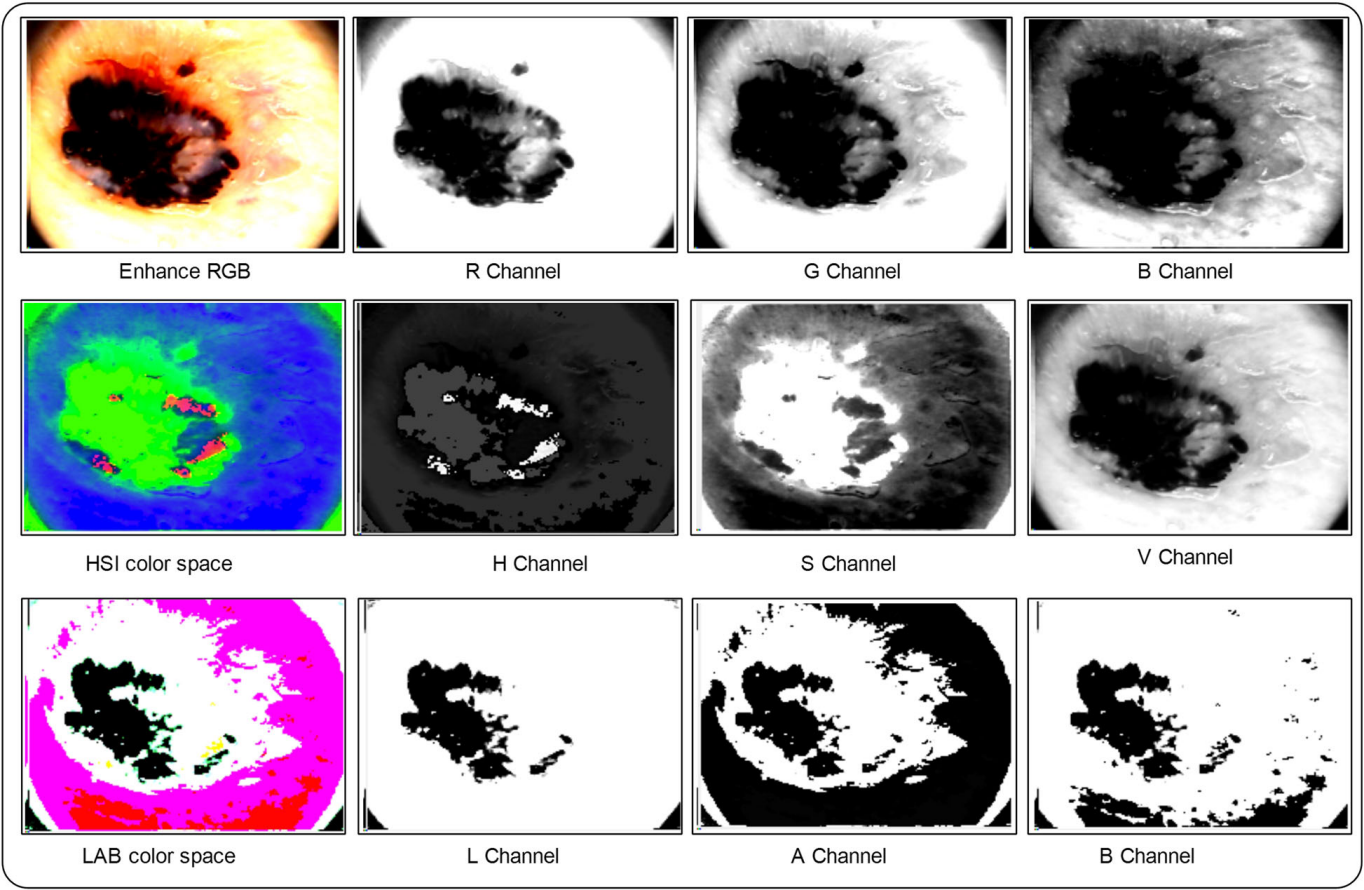
Selected channels for color features extraction

The color information of the region of interest has attained strong prevalence for classification of lesions in malignant or benign. The color features provide a quick processing and are deeply robust to geometric variations of lesion patterns. Three types of color spaces are utilized for color features extraction such as RGB, HSI, and LAB. As shown in Fig. 9, the mean, variance, skewness and kurtosis are calculated for each selected channel. From Fig. 8, its shown clearly that the 1×12 features are extracted from each color space and total features of three color spaces having dimension of 1×36.

#### Features fusion

The goal of feature fusion is to create a new feature vector, which contains much information as compare to individual feature vector. Different types of features are extracted from same image always indicates the distinct characteristics of an image. The combination of these features effectively discriminate the information of extracted features and also eliminates the redundant information between them. The elimination of redundant information between extracted set of features provides improved classification performance. In this work, we implemented a parallel features fusion technique. The implemented technique efficiently fuse the all extracted features and also remove the redundant information between them. The fusion process is detailed as: Suppose *C*_1_,*C*_2_, and *C*_3_ are known lesion classes (i.e. melanoma, atypical nevi and benign). Let $\Theta =\left \{ \psi \ | \ \psi \in \mathbb {R}^{K} \right \}$ denotes the testing images. As given three extracted feature sets $D=\left \{ \alpha \ | \ \alpha \in \mathbb {R}^{h} \right \}, E=\left \{ j \ | \ j \in \mathbb {R}^{t} \right \}, \left \{ o \ | \ o \in \mathbb {R}^{c} \right \}$, where *α*, *j* and *o* are three feature vector (i.e. HOG, texture and color). Then the parallel fusion is define as:

32 $$ F\big(P^{//}\big)=(\alpha_{1}, \alpha_{2}, \ldots \alpha_{d})(j_{1}, j_{2},\ldots j_{d})(o_{1},o_{2},\ldots o_{d})  $$

Where *d* denotes the dimension of extracted set of features. As we know the dimension of each extracted feature vector (i.e. HOG (1×200), Texture (1×42) and Color (1×36). Then the fused vector is define as:

33 $$ \Upsilon \big(F_{s}^{//}\big)=\left (\alpha + \iota \ j, \alpha + \iota \ o \ | \ \alpha \in D, \ j \in E, \ o \in F\right)  $$

It in an *n* dimensional complex vector, where *n*=*max*(*d*(*D*),*d*(*E*),*d*(*F*)). From previous expression, the HOG has maximum dimension 1×200. Hence, make the size of E and F feature vector equally to *D* vector. For this purpose adding zeros. For example below is a given matrix, which consists of three feature vectors.

34 $$ \left\{\begin{array}{l} D= (0.2 \ \ 0.7 \ \ 0.9 \ \ 0.11 \ \ 0.10 \ \ 0.56 \ \ \ldots \ \ 0.90)\\ E=(0.1 \ \ 0.3 \ \ 0.5 \ \ 0.17 \ \ 0.15)\\ F=(0.3 \ \ 0.17 \ \ 0.93 \ \ 0.15) \end{array}\right.  $$

Then make the same size of feature vector, by adding zeros.

35 $$ \left\{\begin{array}{l} D= (0.2 \ \ 0.7 \ \ 0.9 \ \ 0.11 \ \ 0.10 \ \ 0.56 \ \ ... \ \ 0.90)\\ E=(0.1 \ \ 0.3 \ \ 0.5 \ \ 0.17 \ \ 0.15 \ \ 0.0 \ \ ... \ \ 0.0)\\ F=(0.3 \ \ 0.17 \ \ 0.93 \ \ 0.15 \ \ 0.0 \ \ 0.0 \ \ ... \ \ 0.0) \end{array}\right.  $$

Finally, a novel feature selection technique is implemented on fused features vector and select the most prominent features for classification.

### Features selection

The motivation behind the implementation of feature selection technique is to select the most prominent features for improving the accuracy and also make the system fast in terms of execution time. The major reasons behind feature selection technique are a) utilize only a selected group prominent features leads to increased the classification accuracy by the elimination of irrelevant features; b) the miniature group of features is discovered that maximally increases the performance of proposed method; c) select a group of features from the high dimensional features set for a dense and detailed data representation. In this work, a novel Entropy-Variances based feature selection method is implemented. The proposed method performs in two steps. First, it calculates the Bhattacharyya distance of fused feature vector. The Bhattacharyya distance find out the closeness between two features. It is utilized for classification of lesion classes and also consider more reliable as compare to Euclidean distance. Second, it implements an entropy-variance method on closeness features and select the most prominent features based on their maximum values. Entropy in a nutshell is the uncertainty measurement associated with initialization of the closeness features. Since base classifier is highly dependent on their initial conditions for their fast convergence and accurate approximation. Also, the selected closeness features should have maximum entropy value. To the best of our knowledge, entropy, especially in conjunction with Bhattacharyya distance and Variances, has never been adopted for selection of most prominent features. Let *f*_*i*_ and *f*_*i*+1_ are two features of fused vector $\Upsilon \left (F_{s}^{//}\right)$. The Bhattacharyya distance is calculated as:

36 $$ \vec{ B_{d}}=-ln \left(\sum\limits_{u \in \Upsilon \big(F_{s}^{//}\big)}\sqrt{\left(f_{i}(u). f_{i+1}(u)\right)}\right)  $$

Then Entropy-variance is performed on crossness vector to find out the best features based of their maximum entropy value.

37 $$ \begin{aligned} {}E_{V}\left(\vec{ B_{d}}\right) &= -\frac{ln\left(f_{(i+1)}+ \sigma^{2}\right)}{ln\left(f_{i}+\sigma^{2}\right)+ ln\left(f_{i}-\sigma^{2}\right)}\\ &\quad \sum\limits_{f=1}^{\Upsilon} \left(H_{f_{i}}^{0} / \delta H \right) \; log_{2} \left(H_{f_{i}}^{0} / \delta H\right) \end{aligned}  $$

38 $$ \delta H = \sum\limits_{f=0}^{\Upsilon -1}H_{0}^{i}  $$

where $H_{i}^{j}$ denotes the closeness set of features. Hence the size of selected feature vector is 1×172. The selected vector is feed to multi-class SVM for classification of lesion (i.e. melanoma, benign). The one-against all multi-class SVM [42] is utilized for classification.

## Results

### Evaluation protocol

The proposed method is evaluated on four publicly available datasets including PH2, ISIC, and collective ISBI (ISBI 2016 and ISBI 2017). The proposed method is a conjunction of two primary steps: a) lesion identification; b) lesion classification (i.e. melanoma, benign, atypical nevi). The lesion identification results are discussed in their own section. In this section, we discussed proposed lesion classification results. Four classifications three types of features are extracted (i.e. texture, HOG, and color). The experimental results are obtained on each feature set individually and then compare their results with proposed feature vector (fused vector). The multi-class SVM is selected as a base classifier and compare their results with nine classifications method (decision tree (DT), quadratic discriminant analysis (QDA), quadratic SVM (Q-SVM), logistic regression (LR), Naive Bayes, weighted K-Nearest Neighbor (w-KNN), ensemble boosted tree (EBT), ensemble subspace discriminant (ESDA), and cubic KNN (C-KNN)). Seven measures are calculated for testing the performance of proposed method such as sensitivity, specificity, precision, false negative rate (FNR), false positive rate (FPR), and accuracy. Also, calculate the execution time of one image. The proposed method is implemented on MATLAB 2017a having personal computer Core i7 with 16GB of RAM.

### Datasets & results

#### PH2 Dataset

The PH2 dataset [51] consists of 200 RGB dermoscopic images and of resolution (768×560). This dataset has three main divisions; a) melanoma; b) benign; c) common nevi. There are 40 melanoma, 80 benign and 80 common nev image are in this dataset. For validation 50:50 strategy is performed for training and testing of proposed method. Four experiments are done on different feature sets (i.e. harlick features, color features, HOG features, proposed features fusion and selection method) for given a comparison between individual set of features and proposed feature set. The proposed features fusion and selection with entropy-variances method results are depicted in Table 3. The proposed method obtain maximum accuracy 97.06%, sensitivity 96.67%, specificity 98.74%, precision 97.06% and FPR is 0.01. The individual feature set by without utilizing feature selection algorithm results are depicted in Table 4. The results of Tables 3 and 4 are confirmed by their confusion matrix in Table 5, which shows that proposed features fusion and selection method efficiently perform on base classifier as compare to other classification methods. The comparison of proposed method on PH2 dataset also given in Table 6, which shows the authenticity of proposed method.

**Table 3:** Proposed features fusion and selection results on PH2 dataset

Method	Execution time /sec	Sensitivity (%)	Precision (%)	Specificity (%)	FNR (%)	FPR	Accuracy (%)
DT	7	88.33	88.73	92.50	10.0	0.04	90.0
QDA	2	90.83	89.40	91.20	9.0	0.04	91.0
Q-SVM	2	95.83	96.60	98.70	3.0	0.01	97.0
LR	6	92.10	92.76	96.96	6.0	0.02	94.0
N-B	3	89.60	91.73	96.90	7.5	0.03	92.5
W-KNN	2	91.67	92.33	96.20	6.5	0.02	93.5
EBT	5	95.43	96.67	98.12	3.5	0.02	96.5
ESD	10	94.20	94.53	97.50	4.5	0.02	95.5
C-KNN	2	91.26	91.56	95.61	7.0	0.03	93.0
**Multi-class SVM**	**1**	**96.67**	**97.06**	**98.74**	**2.5**	**0.01**	**97.5**

Data in bold are significant

**Table 4:** Results of individual extracted set of features using PH2 dataset

Name	Features			Performance measures					
Classification Method	Harlick	HOG	Color	Sensitivity (%)	Precision (%)	Specificity (%)	FNR (%)	FPR	Accuracy (%)
Decision tree	✓			67.53	67.50	70.05	31.50	0.16	68.5
		✓		71.67	72.1	85.0	23.0	0.11	77.0
			✓	87.93	86.93	86.9	12.5	0.06	87.5
Quadratic discriminant analysis	✓			70.0	68.43	70.0	30.0	0.14	70.0
		✓		74.60	75.83	88.15	20.0	0.09	80.0
			✓	84.6	81.9	80.65	17.0	0.08	83.0
Quadratic SVM	✓			68.33	70.27	76.25	28.5	0.14	71.5
		✓		82.5	83.37	92.7	13.5	0.06	86.5
			✓	93.77	93.33	94.44	6.0	0.03	94.0
Logistic regression	✓			63.36	64.06	70.05	34.0	0.17	66.0
		✓		86.27	85.83	91.9	11.5	0.09	88.5
			✓	89.2	90.43	92.55	9.5	0.04	90.5
Naive bayes	✓			62.9	62.9	66.85	35.5	0.18	64.5
		✓		81.25	81.93	90.65	15.0	0.07	85.0
			✓	87.93	87.63	90.65	11.0	0.06	89.0
Weighted KNN	✓			66.67	67.5	72.5	31.0	0.16	69.0
		✓		81.67	83.27	92.5	14.0	0.06	86.0
			✓	90.87	90.83	92.55	8.5	0.03	91.5
Ensemble boosted tree	✓			68.33	67.77	68.75	31.5	0.16	68.5
		✓		80.67	82.57	91.3	15.0	0.07	85.0
			✓	88.37	89.47	91.3	10.5	0.04	89.5
Ensemble subspace discriminant	✓			68.76	68.4	71.9	30.0	0.15	70.0
		✓		87.1	87.03	91.9	11.0	0.05	89.0
			✓	92.9	94.7	96.9	5.5	0.03	94.1
Cubic KNN	✓			65.43	66.4	71.9	32.0	0.16	68.0
		✓		80.4	80.8	89.4	16.0	0.07	84.0
			✓	90.3	89.83	91.7	9.5	0.04	90.5
Proposed	✓			69.6	72.23	75.65	28.0	0.14	72.0
		✓		86.27	87.37	94.4	10.5	0.02	89.5
			✓	94.6	93.97	94.4	5.5	0.02	94.5

**Table 5:** Confusion matrix for PH2 dataset

Confusion Matrix: Proposed features fusion and selection				
Class	Tested images	Melanoma	Benign	Caricinoma
Melanoma	20	92.5%	7.5%	
Benign	40	2.5%	97.5%	
Caricinoma	40			100%
Confusion matrix: Harlick features				
Class	Total Images	Melanoma	Benign	Caricinoma
Melanoma	20	57.5%	35%	7.5%
Benign	40	8.8%	68.8%	22.5%
Caricinoma	40	3.8%	13.8%	82.5%
Confusion matrix: HOG features				
Class	Total Images	Melanoma	Benign	Caricinoma
Melanoma	20	70%	30%	-
Benign	40	10%	88.8%	1.3%
Caricinoma	40	-	-	100%
Confusion matrix: Color features				
Class	Total Images	Melanoma	Benign	Caricinoma
Melanoma	20	95%	5.0%	-
Benign	40	3.8%	95%	1.3%
Caricinoma	40	1.3%	5.0%	93.8%

**Table 6:** PH2 dataset: Comparison of proposed algorithm with existing methods

Method	Year	Sensitivity %	Specificity %	Accuracy %
Abuzaghleh et al. [26]	2014	-	-	91
Barata et al. [27]	2013	85	87	87
Abuza et al. [43]	2015	-	-	96.5
Kruck et al. [44]	2015	95	88.1	-
Rula et al. [45]	2017	96	83	-
Waheed et al. [46]	2017	97	84	96
Sath et al. [47]	2017	96	97	-
GUU et al. [48]	2017	94.43	81.01	-
Lei et al. [49]	2016	87.50	93.13	92.0
MRastagoo et al. [50]	2015	94	92	-
**Proposed**	**2017**	**96.67**	**98.7**	**97.5**

Data in bold are significant

#### ISIC dataset

The ISIC dataset [52] is an institutional database and often used in skin cancer research. It is an open source database having high-quality RGB dermoscopic images of resolution (1022×1022). ISIC incorporates many sub-datasets but we selected: a) ISIC MSK-2 and b) ISIC-UDA. From ISIC MSK-2 dataset, we collected 290 images having 130 melanoma and 160 benign. For validation of proposed algorithm, we have performed four experiments on different types of features (i.e. Harlick features, Color features, HOG features and proposed features fusion and selection vector). Four different classification methods are compared with the base classifier (multi-class SVM). The proposed features fusion and selection results are shown in Table 7 having maximum accuracy 97.2%, sensitivity 96.60% and specificity 98.30% on the base classifier. The individual feature set results are depicted in Table 8, and base classifier (multi-class SVM) perform well as compared to other methods. The base classifier results are confirmed by their confusion matrix given in Table 9. From ISIC UDA dataset, we select total 233 images having 93 melanoma and 140 benign. The proposed method results are depicted in Table 10 having maximum accuracy 98.3% and specificity 100% on the base classifier. Also, the results on individual feature sets are depicted in the Table 11, which shows that the proposed features fusion and selection method perform significantly well as compared to Table 10. The base classifier results are confirmed by their confusion matrix given in the Table 12, which shows the authenticity of proposed method.

**Table 7:** Proposed features fusion and selection results on ISIC-MSK dataset

Method	Performance measures					
	Sensitivity (%)	Precision (%)	Specificity (%)	FNR (%)	FPR	Accuracy (%)
Decision tree	92.95	93.1	94.30	6.9	0.07	93.1
Quadratic discriminant analysis	95.95	95.45	91.90	4.5	0.04	95.5
Quadratic SVM	96.25	96.10	95.60	3.8	0.03	96.2
Logistic regression	95.10	95.10	95.60	4.8	0.04	95.2
Naive bayes	92.80	93.30	95.60	6.9	0.07	93.1
Weighted KNN	95.10	95.10	95.60	4.8	0.04	95.2
Ensemble boosted tree	95.10	95.10	95.60	4.80	0.04	95.2
Ensemble subspace discriminant	95.10	95.10	95.60	4.8	0.04	95.2
Cubic KNN	89.35	90.65	95.60	10.0	0.10	90.0
Proposed	**96.60**	**97.0**	**98.30**	**2.8**	**0.01**	**97.2**

Data in bold are significant

**Table 8:** Results for individual extracted set of features using ISIC-MSK dataset

Classifier	Selected features			Performance measures					
	Color	HOG	Harlick	Sensitivity %	Precision %	Specificity	FNR %	FPR	Accuracy %
DT	✓			89.4	89.65	0.919	10.3	0.105	89.7
		✓		92.25	93.10	0.944	6.9	0.06	93.1
			✓	80.95	82.15	0.888	18.3	0.18	81.7
QDA	✓			86.05	86.05	0.875	13.8	0.13	86.2
		✓		94.30	93.85	0.894	6.2	0.05	93.8
			✓	70.73	73.25	0.769	26.6	0.26	73.4
Q-SVM	✓			95.6	95.75	0.956	4.1	0.03	95.9
		✓		95.5	95.46	0.956	4.5	0.04	95.5
			✓	82.05	82.3	0.856	17.6	0.17	82.4
LR	✓			92.05	92.7	0.956	7.6	0.07	92.4
		✓		95.1	95.1	0.956	4.8	0.04	95.2
			✓	81.45	82.25	0.875	17.9	0.18	82.1
N-B	✓			90.9	91.8	0.956	8.6	0.08	91.4
		✓		93.95	94.2	0.956	5.9	0.05	94.1
			✓	82.2	83.95	0.913	16.9	0.03	83.1
W-KNN	✓			90.9	91.9	0.956	8.6	0.08	91.4
		✓		93.95	94.2	0.956	5.9	0.05	94.1
			✓	81.15	84.2	0.938	17.6	0.08	82.4
EBT	✓			91.45	91.85	0.994	8.3	0.08	91.7
		✓		93.35	93.4	0.944	6.6	0.06	93.4
			✓	81.45	82.25	0.875	17.9	0.18	82.1
ESD	✓			86.95	88.05	0.931	12.4	0.125	87.6
		✓		95.5	95.45	0.956	4.5	0.04	95.5
			✓	78.0	79.5	0.875	21.0	0.21	79.0
Cubic KNN	✓			93.25	93.5	0.95	6.6	0.06	93.4
		✓		93.15	92.7	0.973	7.2	0.07	92.8
			✓	76.6	76.6	0.788	23.1	0.23	76.9
Proposed	✓			95.85	95.85	0.963	4.1	0.03	95.9
		✓		97.1	96.75	0.963	3.8	0.02	96.2
			✓	82.55	84.7	0.913	16.6	0.13	83.4

**Table 9:** Confusion matrix for all set of extracted features using ISIC-MSK dataset

Class	Total images	Melanoma	Benign
Confusion matrix: Proposed features fusion and selection			
Melanoma	130	99.2%	1%
Benign	160	4.4%	95.6%
Confusion matrix: Harlick features			
Melanoma	130	73.8%	26.2%
Benign	160	8.8%	91.3%
Confusion matrix: HOG features			
Melanoma	130	99.2%	0.8%
Benign	160	5.0%	95.0%
Confusion matrix: Color features			
Melanoma	130	96.2%	3.8%
Benign	160	3.8%	96.3%

**Table 10:** Proposed features fusion and feature selection results on ISIC-UDA dataset

Method	Measures					
	Sensitivity	Precision	Specificity	FNR	FPR	Accuracy
DT	87.25	90.65	97.1	10.7	0.12	89.3
QDA	79.75	88.60	99.3	16.3	0.19	83.7
QSVM	98.05	98.40	99.3	1.7	0.02	98.3
LR	94.8	96.35	99.3	4.3	0.04	95.7
N-B	88.5	91.00	96.4	9.9	0.10	90.1
W-KNN	83.85	91.20	100	12.9	0.16	87.1
EBT	95.2	95.85	97.9	4.3	0.4	95.7
E-S-D	89.6	89.75	92.1	9.9	0.09	90.1
L-KNN	81.7	90.25	100	14.6	0.18	85.4
**Proposed**	**97.85**	**98.60**	**100**	**1.7**	**0.02**	**98.3**

Data in bold are significant

**Table 11:** Results for individual extracted set of features using ISIC-UDA dataset

Method	Features			Performance measures					
	Color	HOG	Harlick	Sensitivity (%)	Precision (%)	Specificity (%)	FNR (%)	FPR	Accuracy (%)
Decision tree	✓			72.75	77.4	90.7	23.6	0.62	76.4
		✓		70.15	69.4	69.3	30.0	0.30	70.0
			✓	86.55	87.35	91.4	12.4	0.13	87.6
QDA	✓			74.04	74.04	79.3	24.9	0.21	75.1
		✓		77.4	88.45	100	18.0	0.22	82.0
			✓	82.65	83.15	87.9	16.3	0.17	83.7
QSVM	✓			73.7	77.25	89.3	23.2	0.73	76.8
		✓		81.35	89.3	99.3	15.0	0.18	85.0
			✓	94.45	95.8	98.6	4.7	0.05	95.3
LR	✓			68.5	68.35	73.6	30.5	0.31	69.5
		✓		78.5	88.9	100	17.2	0.21	82.8
			✓	93.4	94.65	97.1	5.6	0.05	94.4
N-B	✓			69.4	69.95	78.6	28.8	0.30	71.2
		✓		76.7	76.7	81.4	22.3	0.22	77.7
			✓	86.0	89.05	95.7	12.0	0.13	88.0
W-KNN	✓			74.04	77.9	90.0	22.7	0.21	77.3
		✓		80.8	87.15	97.1	15.9	0.17	84.1
			✓	88.55	92.3	98.6	9.4	0.11	90.6
EBT	✓			71.35	71.8	79.3	27.0	0.23	73.0
		✓		80.8	83.8	92.9	17.2	0.17	82.8
			✓	90.5	91.55	95.0	8.6	0.09	91.4
ESD	✓			69.95	71.6	82.9	27.5	0.30	72.5
		✓		60.2	74.5	85.0	24.9	0.27	75.1
			✓	83.9	86.5	93.6	14.2	0.15	85.8
Cubic KNN	✓			71.7	74.4	86.4	25.3	0.23	74.7
		✓		80.15	87.4	97.9	16.3	0.19	83.7
			✓	85.5	90.2	97.9	12.0	0.14	88.0
Proposed	✓			73.65	78.5	91.4	22.7	0.22	77.3
		✓		82.6	87.55	96.4	14.6	0.15	85.4
			✓	95.2	95.85	97.9	4.3	0.04	95.7

**Table 12:** Confusion matrix for all set of extracted features using ISIC-UDA dataset

Class	Total images	Melanoma	Benign
Confusion matrix: Proposed features fusion and selection			
Melanoma	93	95.7%	4.3%
Benign	140	-	100%
Confusion matrix: Harlick features			
Melanoma	93	55.9%	44.1%
Benign	140	8.6%	91.4%
Confusion matrix: HOG features			
Melanoma	93	68.8%	31.2%
Benign	140	3.6%	96.4%
Confusion matrix: Color features			
Melanoma	93	92.5%	7.5%
Benign	140	2.1%	97.9%

#### ISBI - 2016 & 17

These datasets - ISBI 2016 [52] and ISBI 2017 [53], are based on ISIC archive, which is a largest publicly available collection of quality controlled dermoscopic images for skin lesions. It contains separate training and testing RGB samples of different resolutions, such as ISBI 2016 contains 1279 images (273 melanoma and 1006 benign), where 900 images for training and 350 for testing the algorithm. The ISBI 2017 dataset contains total 2750 images (517 melanoma and 2233 benign) including 2000 training images and 750 testing. For experimental results, first experiments are done on each dataset separately and obtained classification accuracy 83.2%, and 88.2% on ISBI 2016, and ISBI 2017, respectively. The classification results are given in Tables 13 and 14, which is proved by their confusion matrix given in Table 16. After that, both datasets are combined and 10 fold cross-validation is performed for classification results. The maximum classification accuracy of 93.2% is achieved with multi-class SVM, presented in Table 15, which is also confirmed by their confusion matrix given in Table 16. The proposed method is also compared with [54], which has achieved maximum classification accuracy of 85.5%, AUC 0.826, sensitivity 0.853, and specificity 0.993 on ISBI 2016 dataset. However, with our method, achieved classification accuracy is 93.2%, AUC 0.96, sensitivity 0.930, and specificity 0.970, which confirms the authenticity and efficiency of our algorithm on combined dataset compared to [54]. Moreover, in [55] reported maximum AUC is 0.94 for skin cancer classification for 130 melanoma images, however, our method achieved AUC 0.96 on 315 melanoma images. In [56] and [57], the classification accuracy achieved is 85.0% and 81.33% for ISBI 2016 dataset. Upon comparison with [54–56], and [57], the proposed method performs significantly better on both (ISBI 2016 & 17) datasets.

**Table 13:** Classification results on ISBI 2016 dataset

Method	Sensitivity (%)	Precision (%)	Specificity (%)	FNR (%)	FPR	Accuracy (%)	AUC
DT	63.0	62.0	79.0	28.5	0.370	71.5	0.63
QDA	68.0	65.5	79.0	26.4	0.320	73.6	0.74
Q-SVM	68.5	78.5	95.0	17.7	0.315	82.3	0.81
LR	67.0	65.0	79.0	26.1	0.330	72.9	0.69
NB	74.5	77.0	91.5	17.1	0.255	82.9	0.84
W-KNN	70.5	75.0	91.0	18.7	0.295	81.3	0.83
EBT	66.0	**80.0**	**97.0**	18.3	**0.034**	81.7	0.79
ESDA	72.5	55.0	90.0	18.5	0.275	81.5	0.83
Proposed	**75.5**	78.0	93.0	**16.8**	0.270	**83.2**	**0.85**

Data in bold are significant

**Table 14:** Classification results on ISBI 2017 dataset

Method	Sensitivity (%)	Precision (%)	Specificity (%)	FNR (%)	FPR	Accuracy (%)	AUC
DT	74.5	75.0	77	25.5	0.255	74.8	0.77
QDA	77.5	78.0	81	22.5	0.254	77.6	0.78
Q-SVM	86.5	86.5	87	13.8	0.135	86.2	0.92
LR	84.5	84.5	86	15.4	0.135	84.6	0.92
NB	79.5	80.0	83	21.5	0.212	79.5	0.80
W-KNN	87.5	88.0	88	12.2	0.125	87.8	0.92
EBT	86.0	83.5	92	14.2	0.140	85.8	0.91
ESDA	83.5	83.5	87.0	16.5	0.165	83.5	0.90
**Proposed**	**88.5**	**88.0**	**91.0**	**11.8**	**0.120**	**88.2**	**0.93**

Data in bold are significant

**Table 15:** Classification results for challenge ISBI 2016 & ISBI 2017 dataset

Method	Performance measures						
	Sensitivity (%)	Precision (%)	Specificity (%)	FNR (%)	FPR	Accuracy (%)	AUC
DT	87.5	88.0	86.0	12.4	0.125	87.6	0.86
QDA	80.0	80.0	79.0	20.0	0.200	80.0	0.86
QSVM	92.5	92.5	95.0	7.4	0.075	92.6	0.95
LR	92.0	91.5	95.0	8.2	0.08	91.8	0.95
NB	92.0	92.5	**97.0**	8.2	0.08	91.8	0.93
W-KNN	88.5	88.5	91.0	11.6	0.115	88.4	0.88
EBT	92.0	92.0	**97.0**	8.3	0.08	91.7	0.95
ESDA	89.5	89.5	91.5	10.4	0.105	89.6	0.94
**Proposed**	**93.0**	**93.5**	**97.0**	**6.8**	**0.07**	**93.2**	**0.96**

Data in bold are significant

**Table 16:** Confusion matrix for ISBI 2016, ISBI 2017, and Combined images dataset

ISBI 2016				
Classs	Classification class		TPR (%)	FNR (%)
Method	Benign	Melanoma		
Benign	**93%**	3%	93%	3%
Melanoma	11%	**53%**	53%	11%
ISBI 2017				
Class	Classification class		TPR (%)	FNR (%)
	Benign	Melanoma		
Benign	**91%**	9%	91%	9%
Melanoma	14%	**86%**	86%	14%
Combined				
Class	Classification class		TPR (%)	FNR (%)
	Benign	Melanoma		
Benign	**97%**	3%	97%	3%
Melanoma	11%	**89%**	89%	11%

Data in bold are significant

## Discussion

In this section, we epitomized our proposed method in terms of tabular and visual results. The proposed method consists of two major steps: a) lesion identification; b) lesion classification as shown in the Fig. 1. The lesion identification phase has two major parts such as enhancement and segmentation. The lesion enhancement results are shown in the Fig. 3, which shows the efficiency of introduced technique. Then the lesion segmentation method is performed and their results in terms of quantitative and tabular in Table 2 and Figs. 4, 5, 6 and 7. After this extract multi-level features and fused based on parallel strategy. Then a novel feature selection technique is introduced and performed on fused feature vector to select the best features as shown in Fig. 8. Finally, the selected features are utilized by a multi-class SVM. The multi-class SVM selected as a base classifier. The purpose of features fusion and selection is to improve the classification accuracy and also make the system more efficient. Three publicly available datasets are utilized for classification purposes such as PH2, ISIC, and Combined dataset (ISBI 2016 and ISBI 2017). The individual feature results on selected datasets are presented in the Tables 4, 8, and 11. Then compared their results with proposed features fusion and selection as presented in the Tables 3, 7, and 10, which shows that proposed method performs significantly better in terms of classification accuracy and execution time. The base classifier results are also confirmed by their confusion matrix given in Tables 5, 9, and 12. Also, the comparison results of the PH2 dataset with existing methods is presented in the Table 6, which shows the efficiency of proposed method. Moreover, the proposed method is also evaluated on combination of ISBI 2016 and ISBI 2017 dataset and achieved classification accuracy 93.2% as presented in Table 15. The classification accuracy of proposed method on Combined dataset is confirmed by their confusion matrix given in Table 16, which shows the authenticity of proposed method as compared to existing methods.

## Conclusion

In this work, we have implemented a novel method for the identification and classification of skin lesions. The proposed framework incorporates two primary phases: a) lesion identification; b) lesion classification. In the identification step, a novel probabilistic method is introduced prior to features extraction. An entropy controlled variances based features selection method is also implemented by combining Bhattacharyya distance, and with an aim of considering only discriminant features. The selected features are later utilized for classification in its final step using multi-class SVM. The proposed method is tested on three publicly available datasets (i.e. PH2, ISBI 2016 & 17, and ISIC), and it is concluded that the base classifier performs significantly better with proposed features fusion and selection method, compared to other existing techniques in term of sensitivity, specificity, and accuracy. Furthermore, the presented method achieved satisfactory segmentation results on selected datasets.

## Abbreviations

ABCD: Atypical, border, color, diameter
ACS: American Cancer Society
CAD: Computer Aided Design
C-KNN: Cubic KNN
DCT: Discrete Fourier Transform
DT: Decision tree
EBT: Ensemble boosted tree
ESDA: Ensemble subspace discriminant analysis
FFT: Fast Fourier Transform
FNR: False negative rate
GLCM: Gray level co-occurrences matrices
HOG: Histogram Oriented Gradients
LBP: local binary pattern
LOG: Laplacian of Gaussian
LR: Logistic regression
M.D: Mean Deviation
MLR: Multi-scale lesion biased representation
PCA: Principle component analysis
QDA: Quadratic discriminant analysis
Q-SVM: Quadratic SVM
RGB: Red, Green, Blue
SIFT: Scale-invariant feature transform
SVM: Support vector machine
W-KNN: Weighted K-Nearest Neighbor
